# Fine needle aspiration biopsy of intraoral and oropharyngeal mass lesions

**DOI:** 10.1186/1742-6413-5-4

**Published:** 2008-03-28

**Authors:** Husain A Saleh, Lewis Clayman, Haitham Masri

**Affiliations:** 1 Department of Pathology, Sinai-Grace Hospital/Detroit Medical Center, Wayne State University, Detroit, Michigan, USA; 2 Department of Oral-Maxillofacial Surgery, W. Beaumont Hospital, Royal Oak, Michigan, USA; 3 Department of Head & Neck Surgery, University of Michigan, Ann Arbor, Michigan, USA; 4 Department of Otolaryngology/Head and Neck Surgery, Oakwood Hospital and Medical Center, Dearborn, Michigan, USA

## Abstract

**Background:**

Fine needle aspiration (FNA) biopsy has been rarely used in oral and oropharyngeal lesions. The goal of this study was to assess the value and accuracy of FNA biopsy in the diagnosis of oral and oropharyngeal lesions particularly in regards to discriminating benign from malignant tumors.

**Methods:**

Sixteen cases of FNA biopsies obtained of various intraoral and oropharyngeal masses or lesions performed at our institution during the eight-year period from 1998 to 2006 were retrospectively reviewed. The aspiration cytologic diagnoses were correlated with the histologic examination of the corresponding resected lesions.

**Results:**

Sixteen cases of intraoral lesions evaluated by FNA biopsies during the period of 1998–2006 were reviewed. The sites of involvement were: lip [1], maxillary sinus [3], pharynx/oropharynx [5], floor of mouth [4], buccal mucosa [2] and peritonsillar area [1]. Patients' age ranged from 30 to 87 with an average of 54 years. Male to female ratio was 1:3. Cytologically, 7 cases were diagnosed as suspicious/malignant, and 9 cases as benign (including 6 benign neoplasm, 1 atypical, and 2 reactive or "descriptive"). Fifteen cases had corresponding surgical resection for histologic examination, of these, 9 cases were interpreted as malignant, and 6 as benign. There were no false positive diagnoses of malignancy on FNA. Two cases were interpreted as benign or atypical cytologically, but were found to be malignant on histologic examination.

**Conclusion:**

FNA biopsy of intraoral and oropharyngeal masses is a valuable procedure for the initial evaluation of various lesions. It provides helpful information about these lesions and avoids hasty or unnecessary surgical biopsy. It is a rapid and relatively noninvasive procedure. Furthermore, aspiration biopsy is an important tool in the diagnosis and management of these lesions, both neoplastic and non-neoplastic, and can be sometimes complemented by ancillary studies for more accurate interpretation. However, its sensitivity in the diagnosis of malignancy is lower than that of histologic samples. This is probably due to the superficial nature and small size of these lesions, the limited space for maneuvering the needle and difficulty in immobilizing the lesion to obtain adequate samples, rather than to interpretation or inherent limitations of the technique itself.

## Introduction

A wide variety of benign and malignant tumors and non-neoplastic lesions can arise in the intraoral and oropharyngeal areas [1,2]. Squamous cell carcinoma is the most common malignancy, and pleomorphic adenoma of minor salivary glands is the most frequently encountered benign tumor, which usually involves the palate [3-9]. Clinically, cancer of this area may present with pain, bleeding or mass lesion. Traditionally, these masses have been evaluated by surgical biopsy or exfoliative cytology. FNA biopsy has proved to be safe, economic, simple and accurate in the evaluation of various organs including the head and neck [10-12]. However, aspiration biopsy studies of the intraoral and oropharyngeal masses are few and limited [13-16].

In this study, we retrospectively reviewed 16 cases of intraoral and oropharyngeal lesions sampled by FNA biopsy with particular attention to the cytologic features of specific tumors, diagnostic accuracy and cytologic- histologic correlation.

## Materials and methods

Sixteen cases of intraoral and pharyngeal masses and lesions that were sampled by FNA biopsies during the period of 1998–2006 were retrieved from the archives of the pathology department of the Detroit Medical Center, Detroit, Michigan. There were 4 men and 12 women with a male: female ratio of 1:3. They had wide age range from 30 to 87 years with an average age of 54 year old. The pharynx- oropharynx was the most common site of involvement (5 cases). Other sites included the maxillary sinus [3], floor of mouth [4], buccal mucosa [2] and lip and paratonsillar (1 for each). The FNA biopsies were performed mostly by the head & neck surgeons with on-site evaluation for adequacy and triage by cytopathologist, cytopathology fellow or experienced cytotechnologist on most cases. The aspirates were performed, consistently and similarly by all the physicians, according to the standard procedure using 22–23 gauge needles connected to a 10 c.c. plastic syringe and sometimes mounted on Cameco aspiration handle (Precision Dynamics Corp., Burbank, California, USA). After localizing and immobilizing the mass, the aspiration needle was passed through the lesion 2 or 3 times in each case. Air- dried Diff-Quik stained smears were made for immediate evaluation, the remaining smears were alcohol- fixed and stained with Papanicolaou method. The syringe and needle were also rinsed in cytofixative and cell blocks were made in 14 cases whenever sufficient material permitted.

Of the 16 FNA cases, 15 had subsequent corresponding surgical specimens, either a biopsy or surgical resection.

## Results

Table 1 shows the demographic, cytologic and histologic findings of all patients. The average patient's age was 54 years (range 30–87). There was an obvious female predilection with a male: female ratio of 1:3 (4 men and 12 women). The distribution of the lesions varied, but pharynx/oropharynx was the most common location [5]. Other sites included: upper lip mucosa [1], left peritonsillar area [1], maxillary sinus [3], floor of mouth [4], and buccal lesions [2]. The most common clinical presentations were pain, bleeding and a mass lesion.

**Table 1:** Summary of all oral/oropharyngeal FNA cases with clinical and cyto-histologic correlation

**Case**	**Age/Sex**	**Location/clinical**	**FNA Dx**	**Histology Dx**	**FNA review Dx**
1	63 M	Upper lip	Suspicious for adenocarcinoma	Adenocarcinoma, NOS	Same
2	87 F	Lt. paratonsillar swelling	Mixed lymphoid population	Reactive lymphoid hyperplasia	Same
3	30 F	Rt maxillary sinus	Benign. Fibrous and epithelial cells	ameloblastoma	Blood, fibrous and epithelial groups
4	48 M	Lt maxillary sinus	Spindle cell tumor, c/w sarcoma	Total maxillectomy LG myofibroblastic sarcoma	Spindle cell tumor
5	47 F	Lt. maxillary sinus	Malignant. c/w carcinoma	Sinonasal undiff. carcinoma	Carcinoma
6	43 F	Lt parapharyngeal nodule	Epithelial/myoepithelial lesion	Metastatic breast ca	c/w carcinoma
7	57 F	Rt. Parapharyngeal mass	s/o granular call tumor	Adult rhabdomyoma	Benign neoplasm
8	47 F	Lt parapharyngeal mass	c/w pleomorphic adenoma	Pleomorphic adenoma	Same
9	45 M	Rt. Lateral oropharynx lesion	Atypical squamous cells	Invasive sq. ca	s/o sq. ca
10	62 F	Parapharyngeal mass	Pleomorphic adenoma	Pleomorphic adenoma	Same
11	55 F	Lt mandible, lingual plate area	s/o LG mucoepidermoid ca	Low grade mucoepidermoid ca	Same
12	40 F	Rt. Mandibular/floor of mouth lesion	c/w granular cell tumor	No surgical specimen	Same
13	81 F	Floor of mouth	Suspicious for squamous carcinoma	Squamous carcinoma	Same
14	39 M	Rt. Tongue/floor of mouth	suspicious for squamous carcinoma	HG dysplasia/squamous carcinoma	Suspicious squamous cells
15	75 F	Lt posterior buccal mass	s/o plasmacytoma	plasmacytoma	Plasmacytoma
16	51 F	Rt. Buccal lesion	Benign. c/w squamous cyst	Benign squamous cyst	Same

**Abbreviations: **F: female; M: male; Rt.: right; c/w: consistent with; HG: high grade; Lt: left; s/o: suggestive of.

Cytologically, nine cases were diagnosed as benign and included: epithelial/myoepithelial lesion [1], granular cell tumor [2], pleomorphic adenoma [2], squamous cyst [1], reactive/descriptive [2], and atypical squamous cells [1]. Of the two reactive/descriptive cases, one was called reactive lymphoid population and was of a swelling of peritonsillar area confirmed histologically to be reactive lymphoid hyperplasia; and the second was called "benign fibrous and epithelial cells" and was of a right maxillary sinus mass confirmed to be unicystic ameloblastoma. The case of right lateral oropharyngeal lesion called "atypical squamous cells" on FNA turned to be invasive squamous carcinoma on surgical resection. On the other hand, seven cases were interpreted as suspicious or malignant and included: adenocarcinoma [1], sarcoma [1], carcinoma NOS [1], mucoepidermoid carcinoma [1], plasmacytoma (!) and squamous cell carcinoma [2].

Histologically, 15 of the 16 FNA cases had corresponding histologic follow up. (Table 1). Of these 6 of 9 cytologically diagnosed benign lesions were also confirmed to be benign (one had no histologic follow up). Two case, however, were found to be malignant (2 false negative FNA cases). one was a left peripharyngeal nodule cytologically called epithelial-myoepithelial lesion and was found to be metastatic breast carcinoma on resection. The second case, lateral oropharynx, was called atypical squamous cells and was found to be squamous cell carcinoma on surgical resection.

Importantly, all 7 cases called malignant on aspiration cytology were also found to be malignant on surgical specimens, with no false positive diagnoses. All of the histologically confirmed benign cases were also diagnosed as benign on FNA cytology (specificity 6/6; 100%). However, as mentioned earlier, we failed to identify two malignant cases on FNA cytology, one was interpreted as benign and one as atypical, which results in sensitivity of (7/9; 77.7%) for malignant diagnosis (2 false negative FNA cases) (Table 2). On further review, these two cases were found to have on-site evaluation done by cytopathology fellow in training.

**Table 2:** Statistical analysis of the 15 FNA cases with corresponding histologic follow up.

	**Histologic Diagnosis**		
	**malignant**	**benign**	**Total**
**FNA Cytologic Diagnosis**	7	0	7
	2	6	8
	9	6	15

True positives = 7, True negatives = 6, False positives = 0 and False negatives = 2.

Sensitivity for FNA cytology diagnosis of malignancy = 7/9 = 77.7%.

Specificity for FNA cytology diagnosis of malignancy = 6/6 = 100%.

### Lymphoid hyperplasia

The FNA smears of the peritonsillar swelling revealed cellular aspirate with singly dispersed mixed population of lymphocytes, plasma cells and scattered histiocytes. Flow cytometry studies confirmed polyclonal lymphoid tissue.

### Ameloblastoma

**Figure 1:**
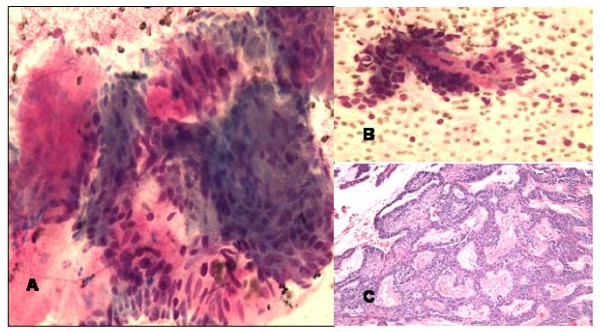
**a and b**. **FNA smears of ameloblastoma of left maxillary sinus showing mixture of palisading groups of epithelial cells with myxoid mesenchymal elements (Diff-Quik, ×400).c**. Histologically, the resected mass showed anastomosing islands and cords of basoloid epithelial tumor cells separated by myxoid mesenchymal stroma (Hematoxylin & Eosin, ×100).

The FNA smears of right maxillary sinus mass revealed palisading groups of epithelial cells admixed with mesenchymal elements. The cells showed low grade atypia, (Fig 1a,b,c).

### Sinonasal undifferentiated carcinoma

**Figure 2:**
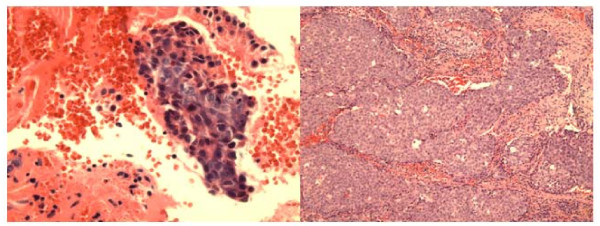
**a**. **Cell block section of FNA of sinonasal undifferentiated carcinoma showing clusters of markedly atypical malignant cells with high nuclear-cytoplasmic ratio, and nuclear hyperchromasia. (Hematoxylin &Eosin, ×400).b**. Histologically, the tumor displays infiltrating large irregular nests of malignant cells with surrounding fibrous stroma. (Hematoxylin & Eosin, ×100).

The FNA smears and cell block showed groups and 3-dimentional clusters of atypical malignant epithelial cells with irregular hyperchromatic nuclei and small nucleoli (Fig 2a,b).

### Pleomorphic adenoma

**Figure 3:**
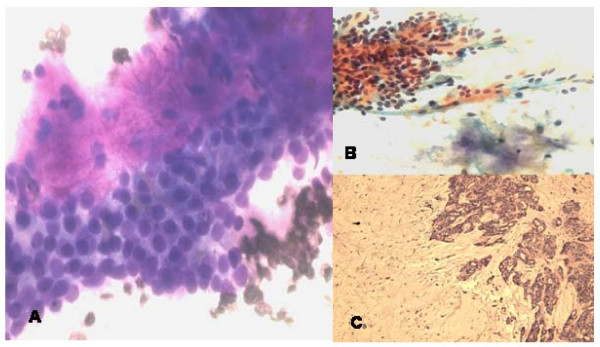
**a**. **FNA smear of minor salivary gland pleomorphic adenoma showing cohesive cluster of uniform low grade epithelial cells with admixed metachromatic myxoid fibrillary stroma and few spindle cells (Diff-Quik, ×400).b**. FNA smear of pleomorphic adenoma showing group of bland uniform epithelial cells and pale fibrillary stroma and few stromal cells (Papanicolaou stain, ×200). **c**. Histologically, islands of epithelial cords and glands and abundant myxoid fibrillary stroma. (Hematoxylin & Eosin, ×100).

The aspirate smears showed biphasic population of epithelial cells and mesenchymal fibrillary myxoid substance. The round to oval epithelial cells were free or mixed within the mesenchymal tissue (Fig 3a,b,c).

**Figure 4:**
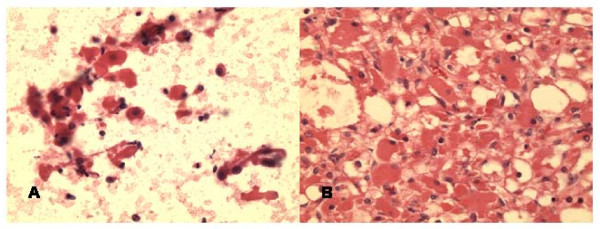
**a**. **FNA smear of adult rhabdomyoma of right peripharyngeal mass showing loose clusters and single large polygonal cells with abundant reddish cytoplasm and round nuclei. Some cells have peripheral nuclei. (Hematoxylin & Eosin, ×400).b**. Histologically, the tumor show diffuse sheets of large cells with abundant reddish cytoplasm and peripheral nuclei (Hematoxylin & Eosin, ×100).

**Figure 5:**
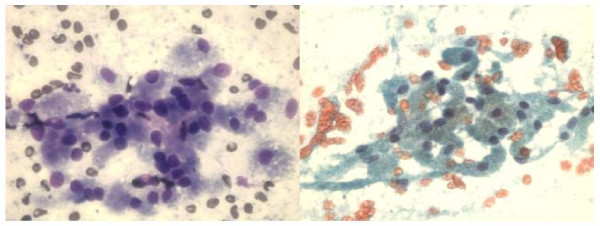
**a and b**. **FNA smears of granular cell tumor displaying discohesive clusters of uniform large cells with abundant granular cytoplasm and central round bland nuclei. (a, Diff-Quik, ×400; b, Papanicolaou satin, ×400).**

The right peripharyngeal FNA case incorrectly interpreted as *granular cell tumor *showed loose clusters or singly dispersed round to polygonal cells with abundant dark eosinophilic cytoplasm and occasional peripheral nuclei (Fig 4a). Interestingly, the resection specimen of this mass revealed adult rhabdomyoma (Fig 4b) that was confirmed by immunostains of myoglobin and myogen (interpretation error of benign tumor). The second FNA case of right mandibular mass showed loose groups of polygonal cells with well demarcated eosinophilic granular cytoplasm and central round nuclei with small nucleoli. The cells were strongly positive for S-100 protein immunostain performed on the cell block confirming the diagnosis of *granular cell tumor* (Fig 5a,b ). 

### Mucoepidermoid carcinoma

**Figure 6:**
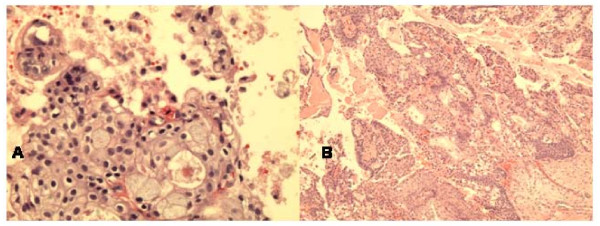
**a**. **Cell block section of low grade mucoepidermoid carcinoma displaying nested squamous cells and glandular cells forming gland with goblet cells (Hematoxylin & Eosin, ×400).b**. Histologically, solid and cystic tumor growth pattern with islands of squamous cells and glandular elements (Hematoxylin & Eosin, ×100).

The FNA smears of the left mandibular area mass displayed mixture of mucous cells (including some goblet cells), squamous cells and few polygonal cells with mucous material in the background. All cells had mild atypia. The cell block section was helpful in revealing fragments with intimate mixture of atypical squamous and glandular cells including goblet cells, consistent with low grade mucoepidermoid carcinoma (Fig 6a,b).

### Squamous cell carcinomas

**Figure 7:**
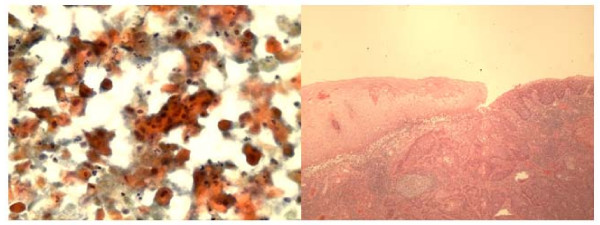
**a**. **FNA smear showing discohesive clusters and single pleomorphic atypical malignant squamous cells with very hyperchromatic nuclei (Papanicolaou stain, ×400).b**. Histologically, invasive islands of squamous carcinoma adjacent to normal squamous mucosa (Hematoxylin & Eosin, ×40).

The aspirate smears showed three dimensional groups and discohesive clusters of atypical squamous cells with varying degrees of keratinization and nuclear atypia. Pleomorphic, hyperchromatic and elongated nuclei were seen, (Fig 7a,b).

**Figure 8:**
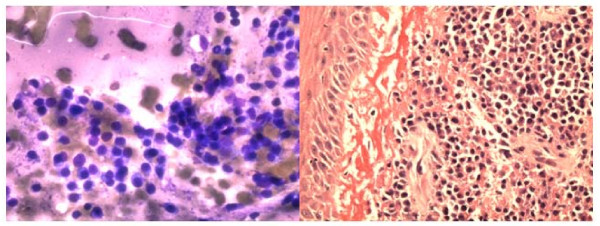
**a**. **FNA smear of plasmacytoma showing monolayer singly dispersed plasma cell. (Diff-Quik stain, ×400).b**. Histologically, diffuse proliferation of atypical plasma cells with overlying benign squamous mucosa (Hematoxylin & Eosin, ×100).

The FNA smears of the left posterior buccal mass showed cellular sample with many somewhat atypical singly dispersed plasmacytes. Size variation and occasional binucleated plasma cells were present. This was diagnosed as *plasmacytoma *and confirmed by immunostains for CD 138, (Fig 8a,b)

The right buccal lesion aspirate displayed many anucleated squames, some spindly or bland benign squamous cells and keratin material consistent with *benign squamous cyst*. The resected specimen also showed retention squamous cyst with benign mature squamous epithelial lining.

## Discussion

The oral and pharyngeal areas can be home for a wide variety of benign and malignant tumors and non-neoplastic lesions. Squamous carcinoma is the most common malignancy [1], but adenoid cystic carcinoma and mucoepidermoid carcinoma of minor salivary glands are also common in this region. On the other hand, pleomorphic adenoma arising from minor salivary glands is the most frequently encountered benign tumor often found in the palate [3-9]. Usually, these patients present with local pain, bleeding or a mass lesion. Surprisingly, many of the patients with oral and oropharyngeal cancers have advanced disease at the time of diagnosis [1,2]. Surgical biopsy is the traditional method for evaluating these lesions but this method may be inconvenient, painful, and costly due to hospitalization and may leave underlying tissue damage. Also, exfoliative or scrape cytology has been attempted, but this method has high false negative rates up to 37%. It was most useful for superficial epithelial lesions but not subepithelial lesions such as salivary gland tumors and lymphomas [2,13]. Over the years, fine needle aspiration (FNA) biopsy cytology has been found to be very useful, simple, cost effective and accurate in assessing and diagnosing various neoplastic and non-neoplastic lesions of many body sites including especially the head and neck [10]. However, the potential, value and accuracy of this procedure in evaluating intraoral and oropharyngeal masses has been studied only rarely, and only a few reports were found in the English literature on this subject [1,2,17-23].

In this study our goal was to investigate the ability of FNA biopsy procedure to accurately diagnose oral and pharyngeal lesions, and to address the cytologic-histologic correlation. We identified 16 cases of this area that underwent FNA biopsy during the period of 1998–2006. Overall, cytologic diagnosis correlated with histologic findings with regards to benign vs. malignant in 13 of 15 cases with accuracy of 86.6% (one case had no tissue). One case of right maxillary sinus was interpreted as "benign, fibrous and epithelial cells" on FNA, and was found to be ameloblastoma on resection (case 3). Another case of right parapharyngeal mass was diagnosed as "suggestive of granular cell tumor" on FNA, but was actually an adult rhabdomyoma on resection (case 7). Importantly, all malignant cytologic diagnoses had a corresponding malignant diagnosis on histology (7 of 7) with no false positive.

Few previous studies addressed the accuracy of FNA biopsy in the diagnosis of oral and oropharyngeal lesions. Scher et al in their study of 93 FNA cases of oral and oropharyngeal lesions, had no false positive diagnoses, but had seven false negative diagnoses [13]. Based on these results and their overall findings, they recommended FNA biopsy for the evaluation of intraoral/parapharyngeal lesions considering its advantages over open biopsy including greater patient comfort, avoidance of general anesthesia, low risk of infection or tissue damage and rapid diagnosis.

In our study, however, 2 cases interpreted cytologically as benign or atypical were actually malignant and later confirmed by tissue examination (false negative diagnoses). The first case of a left oropharyngeal mass revealed what looked like two types of cells: epithelial and myoepithelial suspected to arise from a minor salivary gland. However, the resected specimen showed, surprisingly, metastatic carcinoma of a breast origin. Retrospectively, the cellular pleomorphism (rather than two cell population), immunostains and a history of breast cancer, should have raised the index of suspicion for metastatic breast carcinoma. Finally, the smears of the right lateral oropharynx lesion showed loose discohesive clusters and small irregular tissue fragments of mildly atypical squamous cells. On review, attention to scattered atypical squamous cells with hyperchromatic irregular wrinkled nuclei and considering the presence of a mass lesion may have been good clues to suspect squamous cell carcinoma.

False negative cytologic diagnosis may be due to low cellularity or non-representative sample of the actual lesion. In addition, clinically, some of these lesions are relatively small and superficial making it difficult to reach for precise sampling. They can also be technically difficult to immobilize for making the "back-and -forth" motion in the lesion for ideal aspiration sampling. This emphasizes the significance of correlating the cytologic diagnosis with the clinical history, and if clinical suspicion exists after negative FNA, further investigation should be carried out. Other studies had similar findings and suggested that false negative FNA cytology is most likely related to inadequate specimen or sampling error [15].

One of the malignant cases in our series was a sinonasal undifferentiated carcinoma of the left maxillary sinus and ethmoid recess, which was recognized on FNA as carcinoma, NOS. This rare tumor can be mistaken cytologically with other malignancies and may require immunostains for correct subclassification [12].

FNA biopsy of oral and pharyngeal lesions was shown to be sensitive (93%) and specific (86%) in the study by Shah et. al. Moreover, this technique had advantages over open biopsy with reduced risk of complications such as bleeding, infection and scarring, and ability to identify variety of reactive, benign and malignant lesions [14]. FNA also enables us to triage the specimen during sampling by immediate microscopic evaluation. Cases suspected to be lymphoma or infections can be submitted for flow cytometry evaluation or culture studies, respectively. This triage could help in lowering expenses by avoiding the need for other procedures such as additional samples for culture studies or biopsy. Performing FNA biopsy as initial diagnostic evaluation, rather tissue biopsy, can also save money by reducing in-hospital patient's expenses through eliminating operating room surgical intervention to obtain tissue samples of oral and oropharyngeal lesions.

## Conclusion

In summary, we have demonstrated from our experience of this relatively small series, that FNA biopsy of intraoral and oropharyngeal lesions is a valid procedure and is indeed an important method for the evaluation of these lesions. This technique is simple, financially acceptable and inexpensive, convenient and comfortable to the patient, and above all, can offer a rapid and accurate diagnosis. False negative cases are usually due to inadequate sampling and FNA should be repeated whenever the clinical findings are suspicious for malignancy. Ancillary studies such as immunostains and flow cytometry can greatly help in further classification of the tumors.

## Competing interests

The author(s) declare that they have no competing interests.
